# What’s Preventing Us to Get More Attraction: The Fear of Aesthetic Surgery

**Published:** 2016-09

**Authors:** Mona Leitermann, Klaus Hoffmann, Erich Kasten

**Affiliations:** 1 Medical School Hamburg, University of Applied Sciences, Hamburg, Germany;; 2 Klinik für Dermatologie und Allergologie der Ruhr Universität Bochum, Bochum, Germany;; 3 Department of Neuropsychology, Medical School Hamburg, Hamburg, Germany

**Keywords:** Plastic surgery, Operation, Aesthetic, Beauty, Cosmetic surgery, Fear

## Abstract

**BACKGROUND:**

Nowadays, with the help of cosmetic surgery almost every woman and man can achieve a highly attractive appearance. The question is, why so many people do not take advantage of these opportunities? This pilot-study investigates individual attitudes of people towards aesthetic plastic surgery.

**METHODS:**

A questionnaire was developed which combined self-developed items for a measurement of attitudes towards plastic surgery. In addition, items of the “Freiburger Personality Inventory” (FPI-R) were used. The study was conducted in Hamburg/Germany. 104One hundred and four test persons participated in the survey (81 females, 23 males, age 20-30 years). Eighty six of the participants (82.7%) had an A-level as degree of education, 14.4% achieved the secondary school certificate and 2.9% had completed their bachelor on a high school.

**RESULTS:**

The data supported the hypothesis that people who are unsatisfied with their body appearance showed more willingness for a surgical intervention. On the other hand, fear of complications and pain as far as anxiety before an unsatisfactory result hinders them from a decision for an intervention. Significant correlations with regard to extraversion-introversion and the education level were not found. Females showed more willingness regarding an intervention than men. Gender-specific differences concerning the cost factor were not found.

**CONCLUSION:**

Interestingly more than 65% of the total sample felt dissatisfaction with a specific body part and are thus target of aesthetic surgery. The yellow press often reports about failed cosmetic surgery, especially in VIP-persons. Aesthetic surgery should keep working to reduce unwarranted fears of people toward these kinds of operations.

## INTRODUCTION

Plastic surgery (from “plastein” [greek]=to form) is a section of surgery, which deals with reconstructive operations after deformations, accidents or due to aesthetical causes. Consequently, the plastic surgeon executes corrections of norm deviations or aging processes.

In Germany, the number of aesthetic-surgical interventions is currently about 400.000 per year and is increasing.^1^ In 2013, a representative survey of the German Society for Aesthetic Plastic Surgery e.V. showed a decreasing age; patients between 31 and 50 years were the largest group (45%). The number of patients with an age of more than 50 years was a quarter of all patients in plastic surgery.^1^

In 2011, only 1.3% of the operations were performed in adolescents. Breast enlargement (mamma-augmentation) is the most popular intervention in young women; while it is lid lifting (blepharoplastic) in men. Plastic surgery is required by several patients after injuries or cancer operations,^2^ e.g. breast implants after mastectomies (removal of mammary gland), rhinoplastics (nose corrections) and interventions after septum deviations (nasal septum distortions). Currently, in comparison with the previous years the percentage of males is increasing slightly.^3^ Nowadays, nearly everybody is interested to increase the attractivity of the own appearance. An exciting question is, why claims not every person, who is dissatisfied with her or his appearance, for a plastic surgery? In regard to this question, the role of costs for an aesthetic-plastic treatment is important. The survey of the DGÄPC (2013) showed an ambivalent picture. For the majority of the interviewees costs played a “more than important” role. Approximately two thirds of females and 60 % of males decided against such an intervention due to financial aspects. In contrast, a considerable part of the patients agreed to drive long distances to be treated by a specialist of their choice, which is, in turn, associated with expenditures of time and costs.^3^^,^^4^

A nearly perfect appearance has increasing importance for success in partnership as well as in business. Especially women feel a huge uncertainty towards their own corporeality.^5^^-^^7^ In 2007, the Austrian Gallup-Institute performed a psychological interview with 100 persons in the age between 25 and 60 years, hereof one third were males and two third females. The interview contained questions about the attitudes in regard to cosmetic operations and the connection between appearance and success. 47% of the participants were interested to have a cosmetic-surgical operation.^5^^-^^7^

Two arguments for a cosmetic intervention achieved more than 50% consent: “Well appearance increases the self-confidence” and “Those who look good, feel well”. Nevertheless, 38% of the participants were convinced to find a new partner easier with increased beauty. The results from a survey of the Forsa-Institute (2009) in more than 1,000 girls and women, showed that 68% of the adults were unsatisfied with their appearance and about a quarter of all girls would say “yes” to a cosmetic operation if this would not be associated with costs.^8^

The adolescents of this study were in an age between 11 and 17 years, generally they tended to evaluate their appearance as critically. According to the results of this study, “tenfour” is the most popular intervention: abdominal streamlining, liposuction, breast reduction as well as breast enlargement. Moosavizadeh *et al.* investigated in 2012 seventyfive patients. Rhinoplasty (52%) and abdominoplasty (8%) were the most and least common performed surgeries, respectively. The major important motivators were family, friends, classmates, and colleagues (40%) and the least were magazines and journals (4%).10.7% of the participants described the posture and function of the target organs as perfect, but they planned to improve its aesthetic or functional aspects by surgery.^9^

The opinions of the society in regard to “beauty surgery” are very different. What is the motivation and which reasons give people for the individual attitudes concerning cosmetic surgery? What may persuade people to go for such an intervention? Why will others not even take an intervention into consideration?

## MATERIALS AND METHODS

The here presented study investigated which factors influence the individual attitude towards plastic surgery and what discourages young people, who are unsatisfied with their body, to go for a cosmetic intervention. Main method was the development of a questionnaire to investigate the motivations towards cosmetic surgery and the connection to psycho-social aspects. At first, the optimum sample size (i.e. how many test participants are needed to find significant differences) was calculated.^10^

This calculation delivered an optimum of n=96. In the next step two questionnaires were combined: (i) the Freiburger Personality Inventory^11^ and (ii) self-developed items about the attitude towards plastic surgery. Here, three areas of subscales were formed: a) Individual perceived attractivity; b) Willingness for an operation; c) Specific fear of cosmetic interventions. For the response a numerical rating scale (Likert Scale) was applied; here the participants could select their answer on scale between -50=”I disagree” and +50=”I agree”. To test the reliability of the self-created part of the questionnaire, some nearly identical items in different parts of the questionnaire were developed. These two questionnaires were published via the internet based program “SosciSurvey” (www.soscisurvey.de). After achieving the calculated sample the data were analyzed with the statistic software “SPSS”.

The following hypotheses were created: H1: “The higher the dissatisfaction with a certain physical appearance, the higher the willingness to have an operation.”The background of this hypothesis is the assumption that behind dissatisfaction with specific body parts stands a psychological strain. A cosmetic surgery could reduce this strain and therefore influence the willingness to have an operation. Questions in the here presented study were: (i) I am satisfied with my physical appearance, (ii) I am interested in a cosmetic intervention, (iii) There is an area on my body I’m interested in an operation, (iv) I find myself attractive, and (v) I am dissatisfied with my appearance.

H2: “The greater the people`s fear of complications, the less improbable is the decision for an intervention.” Behind this hypothesis stands the assumption that headlines of mass media about complications and risks of plastic intervention influences the personal attitude towards plastic surgery negatively. Questions were: (i) I am afraid of cosmetic interventions, (ii) If I am unhappy with a certain physical appearance, possible complications of an operation are irrelevant for me, (iii) The fear of complications hinders me to agree in such a cosmetic operation, and (iv) Possible embarrassing consequences of a cosmetic intervention do not stop me to decide for an intervention. 

H3: “The greater the people’s fear of pain, the less improbable is the decision for an intervention.” The fear of pain often stems from negative reports in the yellow press. The more these articles report about strong pains after a cosmetic intervention, the more the individual attitude towards plastic interventions is influenced negatively. Questions were: (i) The fear of post-operative pains hinders me to decide for a cosmetic operation, (ii) I accept possible pains of an intervention to be satisfied with my appearance completely, and (iii) I do not fear the pains, which are related to a cosmetic intervention.

H4: “The greater the people`s fear of possible dissatisfaction with the cosmetic result, the less improbable is an intervention.” This hypothesis also stems from the background that negative headlines about cosmetic operations with embarrassing consequences could cause a deterrent effect in the society about this kind of surgery. Questions for this hypothesis were: (i) I am afraid of being unsatisfied with the result even if the medical part of the operation was successful, (ii) I assume to be satisfied with the result of the cosmetic intervention, (iii) I do not have fears of being unsatisfied with the result of a cosmetic intervention, and (iv) I do have a huge fear of mistakes in the cosmetic intervention.

H5: “Extraverted persons tend considerably more to go for an operation.” The background of this speculation was the assumption that the characteristics of extraverted persons (sociable, mixwell, talkative, active, enthusiastic, vigorously) affect positively the individual attitude towards cosmetic surgery and that extraverted people are consequently more willing to undergo a cosmetic operation if they suffer under a certain physical appearance. For the acquisition of the personality features “extraversion/introversion” the “Freiburger Personality Inventory” (FPI) was applied.

H6: “Women are more willing to undergo an operation than men.” H6a: “Beauty is more important for women than for men.” These hypotheses accrued out of the conjecture that women are more interested in their appearance than men. Among women consists a more competitive pressure due to mutual comparison of physical beauty as well as a higher responsiveness for the own attractivity due to the influence of mass media. Questions were: (i) I am interested in a cosmetic intervention, and (ii) There is an area on my body for which I would undergo a surgical intervention. For the answer of the hypothesis 6a two open questions are formulated in which the participants could indicate, without a given price range, how much they would pay for a nose operation and a liposuction.

H7: “The higher the education level of a person is, the higher is the willingness to go for an intervention.” This thesis leans on the assumption that the education level plays a considerable role for important decisions. Furthermore, it could be assumed that people with a higher education level deal more critical with their physical appearance because they are ranged mainly in the upper social classes. Here a higher competitive pressure exists in regard to individual attractiveness, which is seen as a guarantee for social rise. For the acquisition of this data, socio-demographic information about the achieved degree of education was asked.

H8: “The higher the anticipated costs for the desired operation, the smaller the probability that the patient undergoes an operation.” The background of this speculation is the fact that aesthetic interventions are mostly connected with high costs and often not an urgent need exists (as long as no heavy psychological strain exists). For the investigation of this hypothesis open questions related to the costs of an operation were asked. 

According to the usual interpretation of the inner consistency a value of more than .70 is acceptable, a value above .90 is excellent.^1^ For data analysis the original scale of SosciSurvey from -50 up to +50 was transformed into a scale of 1 to 11 (i.e. the data 6 expresses the average). Data were analyzed with the SPSS program, significant differences were investigated on a level of α=0.05 with non-parametric tests.

## RESULTS

Ultimately 104 test persons participated in the survey (81 females, 23 males). 86 of the participants (82.7%) had the A-level as the degree of education, 14.4% of the participants achieved the secondary school certificate and 2.9% had completed their bachelor on a high school. All participants of this study were young adults (20–30 y.). For analyzing the inner consistency, the Cronbach Alpha was calculated for the three different subscales. From the attractivity-part of the questionnaire (H1: items 1, 4, 5) a value of .90 results. From the part about the individual willingness for a cosmetic operation (H1: items 2 and 3) a value of .91 was found, and for the part that measured the individual fear before plastic surgery (H3 to H5) a value of .79 was calculated. 

**Fig. 1:**
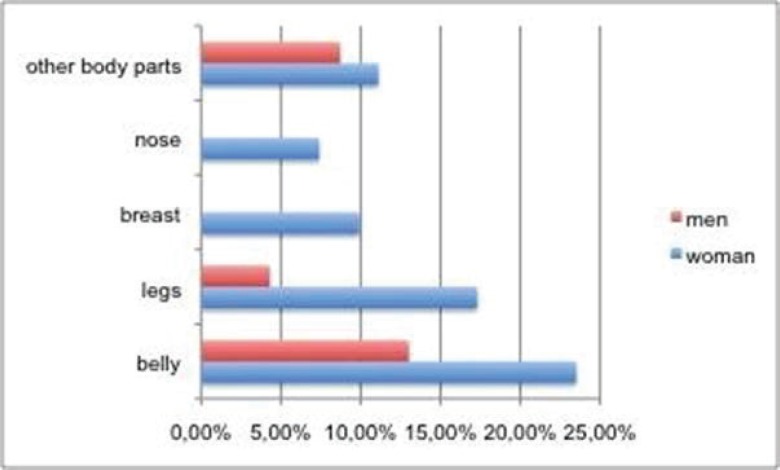
Dissatisfaction with certain body parts, divided into gender (men: n=23, woman: n=81). The percentages are given in regard to the total sample as 100%.

The participants were interrogated, whether dissatisfaction with a particular body part exists? From the 23 male test persons, 73.9% answered to be satisfied with their appearance, 13% found their belly disturbing, 8.7% selected the opinion “other body parts” and 4.3% of the men indicated a dissatisfaction with their legs. 23.5% of the 81 female participants felt a dissatisfaction with their belly, 17.3% were not satisfied with their legs. 11.1% selected the category “other body parts”. The dissatisfaction with breasts was 9.9%, 7.4% found their nose disturbing. In figure 1, these answers are shown. In summary 34.6% of the 104 participants expressed a satisfaction with their body in total and the majority (65.4 %) indicated a dissatisfaction with one or more body parts. 73.9% of men were satisfied with their appearance in total, but only 23.5 % of the women. Both, in female and in male participants, the body zone ‘belly” was indicated as a major problem (20.2 %) and the dissatisfaction with legs was in the second position for women and men, equally with 14.4 %.

Hypothesis H1: “The higher the dissatisfaction with a certain physical appearance, the higher the willingness to have an operation.” To investigate this hypothesis, six Spearman’s Rho correlations were calculated (Table 1). For the correlation between the items „I am satisfied with my physical appearance“ and “I am interested in a cosmetic intervention” a negative connection of R=-0.283 was found. This result was significant (*p*<0.05). The correlation of the items „I am satisfied with my physical appearance“ and “There is a zone on my body, for which I would undergo an operation“ resulted in a correlations coefficient of R=-0.242. 

**Table 1:** Correlations and levels of significance for Hypothesis H1. A correction of the significance levels via Bonferroni correction in multiple testing (Bortz & Döring, 2006)^1^ leads to a border value of 0.008, therefore three of the correlations (b, c, d) are not marked as significant

**Item**	**Correlation coefficient**	**Significance**
“I am satisfied with my physical appearance” and “I am interested in a cosmetic intervention”	-0.283	0.004*
“I am satisfied with my physical appearance” and “There is a zone on my body, for which I would undergo an operation”	-0.242	0.013
“I find myself attractive” was correlated with the item “I am interested in a cosmetic intervention.”	-0.235	0.016
“I find myself attractive” and “There is an area on my body, I’m interested in an operation.”	-0.221	0.024
“I am dissatisfied with my outward appearance” and “I am interested in a cosmetic intervention”	0.304	0.002*
“I am dissatisfied with my physical appearance” was correlated with: “There is an area on my body, I’m interested in an operation.”	0.271	0.005*

This result was significant (*p*<0.05). The item ‘I find myself attractive’ was correlated with the item ‘I am interested in a cosmetic intervention’.“ The calculations showed a significant negative correlation coefficient of R=-0.235. The correlation of the items: ‘I find myself attractive’ and ‘There is an area on my body’ I’m interested in an operation.” resulted in a likewise significant positive connection of R=-0.221. A correlation of the items: ‘I am dissatisfied with my outward appearance’ and ‘I am interested in a cosmetic intervention’ showed a significant correlation coefficient of R=0.304, thus describes a mild to moderate linear, positive connection. The item ‘I am dissatisfied with my physical appearance’ was correlated with: ‘There is an area on my body, I’m interested in an operation’. Here, the calculations resulted in a significant correlations coefficient of R=0.271. In summary, on the basis of these results, the hypothesis H1 appears to be valid.

Hypothesis H2: “The greater the people`s fear of complications, the less improbable is the decision for an intervention.” To analyze the connection between fear of complications and the decision against a plastic surgery the above mentioned four items were used. These items were correlated with the willingness to undergo an operation. The resulting correlations coefficient of R=-0.326 was significant (*p*<0.05) and therefore proved the hypothesis H2 (Table 2).

**Table 2:** Corrrelations and signifcance test (Hypothesis 2-4)

**Hypothesis**	**Correlation with willingness for operation**	**Significance** **(2-sided)**
Hypothesis 2	R=-0.326	0.001
Hypothesis 3	R=-0.486	0.001
Hypothesis 4	R=0.300	0.002

Hypothesis H3: “The greater the people’s fear of pain, the less improbable is the decision to go for an intervention.” The above mentioned three items were correlated with the willingness to undergo an operation. The result of this calculation was a significant negative connection of R=-0.486. Therefore the hypothesis H3 could be proved statistically (Table 2).

Hypothesis H4: “The greater the people’s fear of possible dissatisfaction with the cosmetic intervention, the more improbable to undergo an operation”.“ The above mentioned items were correlated with the willingness to undergo an operation. The calculations delivered a significant connection of R=.300, i.e. the hypothesis H4 is also valid (Table 2).

Hypothesis H5: “Extraverted persons tend more often to go for an operation”. For an investigation of this hypothesis, the sum of the items for extraversion/introversion of the Freiburger Personality Inventory was correlated with the willingness to undergo an operation. The calculations delivered a weak, positive linear connection of R=0.030. Thus, the alternative hypothesis H5 could not be proved, the H0 is accepted.

Hypothesis H6: “Women are more willing to undergo an operation than men”. To investigate this assumption the frequencies of the item “I am interested in a cosmetic in an intervention” were counted. This shows that the majority of women (55.5%) tend in the direction of a “disinterest”. Only two women (2.5%) answered neutrally and 34 of 81 women (41.9%) selected values in the positive area of the scale, which emphasized an interest in a cosmetic intervention. In comparison, the statements of men regarding an interest are in the negative area of the scale. One test person is neutrally and only 17.4% are in the positive area of the scale. To investigate the difference between men and women the Mann-Whitney-U-Test was carried out for the item „I am interested in a cosmetic intervention” (Table 3).

**Table 3:** Results of the U-Test comparison: Interest in a cosmetic intervention between men and women (hypothesis 6, item 17, 18).

**Item**	**Male** **(n=23)**	**Female** **(n=81)**	**U-test** **Significance (** ***p*** ** value)**
I am interested in a cosmetic intervention	37,91	56,64	0.006
There is an area on my body for which I would undergo a surgical intervention	41,65	55,58	0.045

The result of the U-test showed a significant difference between men and women with regard to the interest in a cosmetic intervention. For the item “There is an area on my body for which I would undergo a surgical intervention” 43 of 81 women (53.1%) were in the positive area, 35 of 81 women (43.2%) were in the negative area of this scale; only 3.7% of the women chose the neutral value. 69.6% of the men were in the negative area and 30.4% in the positive half of this scale.

Hypothesis 6a: “Beauty is more important for women than for men.” For two typical operations (nose intervention and liposuction) nine price ranges and the category “no amount” were given (the latter for participants who would not spend any money for an intervention, even if they were unsatisfied with these body parts). For the investigation, whether women would spend more money than men, a Mann-Whitney-U-Test was calculated. The calculations delivered no significant difference for the gender, i.e. the hypothesis H6a could not be proved. There is no significant difference between men and women concerning the amount of the costs, which they would spend for such an operation. Looking at the distributions of the price ranges, there was no clear result, women chose more often the lower price ranges than men, but the distribution was bi- and trimodal for both genders. The following graphs show the given answers (Table 3).

Hypothesis 7: “The higher the educational level of a person, the higher is the willingness to go for an intervention.” Here only a tendency can be shown, because the majority of the participants (82.7%) indicated A-levels as the educational degree, only 14.4% of the test persons achieved the secondary school certificate and 2.9% of the survey participants completed the bachelor on a high school. The majority of high-school graduates (65.1%) express a dissatisfaction with a body part. 19.8% of them specify a dissatisfaction with the body zone “belly”. 15.1% of the high-school graduates sense their legs as a problem zone (Figure 1). 30 test persons with the A-levels (34.9%) specify in contrast a satisfaction with their physical appearance. 5 study participants of the few secondary school graduates (33.3%) express a satisfaction with their physical appearance, 26.7% also indicate the “belly” as a problem zone. The three bachelor graduates distribute dissatisfaction with buttocks, breasts and a satisfaction with the body. For a review of this hypothesis a comparison, how many of the 56 A-level graduates (65.1%) also indicate an increased willingness to undergo an operation, was performed. The result indicated that half of the 56 dissatisfied persons show a willingness to beautify something operatively on their body.

**Fig. 2:**
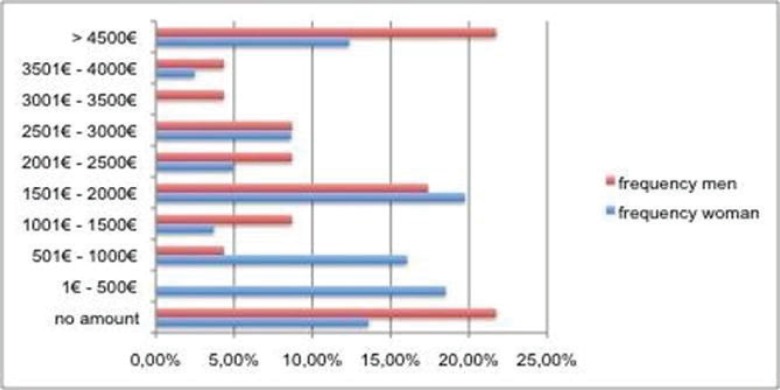
Anticipated price ranges for a nose correction for female (n=81) and male (n=23) participants

Hypthesis H8: “The higher the costs for the desired operation, the lower the probability that a patient undergoes an operation.” For a nose operation 16 of the 104 test persons (15.4%) refused to spend any amount for such an operation. 15 (14.4%) found an amount up to 500.- € as appropriate, 14 test persons (13.5%) felt ready to spend an amount between 500.- € - 1,000.- €. For 20 participants (19.2%) an amount between 1,501.- € and 2,000.- € was it worth and 8.7% of the participants feel ready to spend an amount between 2,500.- € and 3,000,- €. 15 test persons agreed to spend an amount of more than 4,500.- € (14.4%) (Figure 2).

**Fig. 3:**
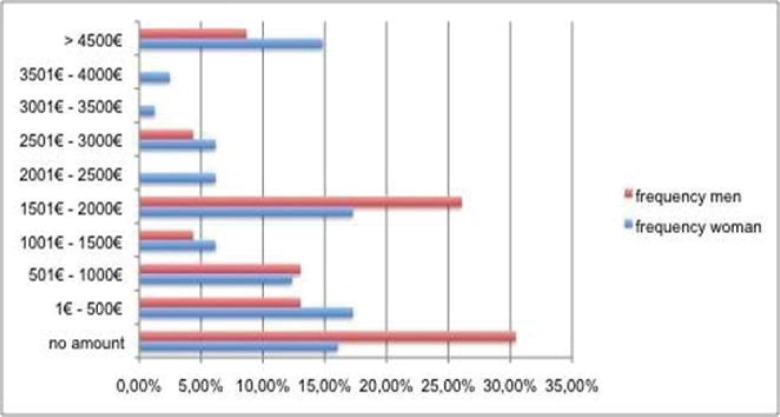
Anticipated price ranges for a liposuction for female (n=81) and male (n=23) participants

For a liposuction 16 test persons (15.2%) refused to spend any amount for such an operation. Fifteen agreed to spend an amount up to 500.- € (14.3%). 20 persons said to spend an amount between 1,500.- € and 2,000.- € (19.2%) and equally, as for the intervention at the nose, 15 test persons were willing to spend an amount of more than 4,500.- € for a liposuction (14.3%) (Figure 3).

For the final analysis of the assumption whether the amount of the respective costs of a surgery stands in connection with the willingness to undergo an operation, the Kruskal-Wallis-Test was performed for each intervention “nose operation” and “liposuction”. The result of this calculation was that due to the non-significant result neither the amount of the costs for an intervention on the nose nor the liposuction stand in connection with a willingness to undergo an operation.

## DISCUSSION

The goal of the here presented study was to investigate the attitudes of people towards plastic surgery and which factors have an influence on the decision for or against a cosmetic intervention. In a century of high autonomy for the appearance of one’s own body,^12^ the demand for aesthetic surgery and body contouring is rapidly increasing.^13^ Interestingly more than 65% of the total sample (n=104) felt a dissatisfaction with a specific body part. In the western culture we have an ideal of active, dynamic youth and aged people try to look younger than they are and the number of wrinkle treatments increases in Germany every year. But the participants of this study were not old; they were young adults (20–30 y.). 

Perhaps this group deals very critical with their appearance. This assumption is proved by a study of the German Society for Aesthetic Plastic Surgery which showed that the largest proportion of cosmetic operations fell in the group of the 18-30 years old.^3^ Even in an older study by the Forsa Institute (2009) it was found that the most cosmetic treatments in Germany are within the age group between 20 and 30. Only 37.4% of the participants of the here presented survey expressed a total satisfaction with their physical appearance, but thereof are still 17 (of 23) men. This high dissatisfaction of the female participants could be based on the fact that we currently have an unreachable ideal of body shape. While in the 1950`s, after starvation in the World war II, for women more round types of the figure were desired, today a slenderness ideal dominates, that included almost all body zones.^14^

The change of the gender roles in the last decades of years could also influence the relationship of women and men to their own body. Nowadays women often penetrate male domains and are confronted with several social expectations. Several studies have shown that attractiveness and success in business stand in a close connection. For example, this was proved by a survey of the Leuphana University Lüneburg in which 3,000 persons were investigated about their income, business success and attractiveness. According to the results of this survey attractive women and men were significantly rarer unemployed and earned more money than their “un-beauty” colleagues.^15^

Furthermore, the high dissatisfaction with the own appearance of women may stand in connection with the media presence. In television, on posters, magazines or on internet site – ideals of beautiful bodies are presented everywhere. Fashion is presented on slim women, diet products are advertised as “miracle cure” for a wonderful figure and women-magazines give tips to lose weight. This may have the consequence that especially the own body awareness of young women is distorted and the required beauty ideals of the society are a continuous pressure.^3^

In one hypothesis of this study the influence of dissatisfaction with a certain physical appearance on the willingness to undergo an operation was investigated. The results showed a moderate but still significant correlation between both variables. This is not astonishing, but the moderate correlation shows that the willingness for an actual intervention depends not only on the dissatisfaction. Other influences as e.g. financial interests, mental strain with the appearance, opinions of the surrounding toward beauty surgery are important as well. The German Association for Aesthetic Plastic Surgery showed that there is a relatively long reflection period between the first considerations and the actual operation. On average here between 6 and 8 years passed, until the actual treatment was performed.^3^

Every surgical treatment is associated with risks. Therefore the anxiety of a person for complications of the surgery has another important influence. The second hypothesis showed that the probability to undergo a cosmetic intervention is lower, if a strong fear exists. Even people with heavy strain due to their appearance, have nevertheless a fear of the possible complications. Nowadays, before such an operation, most people made an internet research^16^ and read medical texts about such risks. A survey on behalf of the Federal Institute for food and agriculture^17^ found that every fifth cosmetic operation in Germany is associated with complications (22% women, 8% men). 

In addition, illness after a beauty surgery founded no claims to the health insurance system and in the case of restrictions in the ability to work, no claims for sickness benefit. These facts influence the decision of people negatively, who take a cosmetic operation into consideration.^18^ Fear of pain is another variable which influences the decision for or against a surgical intervention. The greater the people’s fear of pain, the more improbable is that he/she undergoes an operation. But the here presented data showed only a statistical moderate correlation, which is understandable in times of medical pain killer. So the risk of pain is only a weak additive factor. Another fear is that of possible dissatisfaction with the cosmetic result. This hypothesis was proved statistically with another moderate but significant correlation. Particularly the yellow press media had increased in the last decades of years with headlines about failed cosmetic operations the fear for distortions due to aesthetic surgery.

Has the personality an influence on the decision? The hypothesis was that extraverted people tend more often to undergo an operation, because they are less anxious, go out more often on parties and therefore their attractiveness is much more important than for introverted people. The calculations for this hypothesis resulted in a non-significant correlation coefficient, i.e. the personality characteristic “extraversion” does not stand in a close connection with the willingness to undergo a cosmetic operation. An explanation for this unexpected result could be that extraverted people possess also a high self-esteem and they do not care, if anything on their body was not as attractive as it should be. Furthermore, it can be supposed that extraverted people are less critical with their physical appearance than introverted persons.

Another question of this survey was, whether women are more willing to undergo an operation than men. The results for this hypothesis show that the majority of the interviewed women felt dissatisfaction with a specific body part and that they tend more for undergoing a cosmetic operation (53.1%) than men. In contrast 69.6% of the male participants were not willing to change a body part operatively. The difference was significant. One more argument that could suggest that women are more willing to undergo a cosmetic operation is the mainly female participation of this survey (77.9 %). These results match the survey of the German Association for Aesthetic Plastic Surgery in which 1,134 participants in Germany were investigated. Here the proportion was female 82.9%: 17.1% male.

Another hypothesis of this study was that for women beauty is more important than for men. This hypothesis was not proved to be right; between women and men was no significant difference with regard to the anticipated costs which were accepted for an aesthetic operation. The amounts which the participants of this study would spend for a cosmetic operation varied considerable. The female persons answered they would spend about 2,000.- € for a nose correction as well as for liposuction, while men in average would accept even higher costs. According to the German Association for Aesthetic Plastic Surgery one can record an increase of male patients annually (2011: 16.3%, 2012: 16.8%, 2013: 17.1%). An investigation about the price of 5,237 clinics and 10,473 doctors the average costs for a nose correction in Germany are on average 2,912.- € and for a liposuction 2,526.- €.^19^ Thus the actual costs of the interventions are underestimated minor by the female test persons and overestimated by the male participants.

Another question of this survey was whether the educational level has any influence on the willingness to undergo an operation. The result of this hypothesis allows only limited interpretation because the majority of the analyzed sample had an A-level graduate (82.7%) and the group with a lower graduate was too small. The fact that 56 persons with A-levels (65.0%) indicated dissatisfaction with their body could be reasoned by the fact that most participants were late adolescence/young adults, an age in which the appearance plays a bigger role than in other phases of life. Especially in the late adolescence beauty is a guarantee for better social connections and easier contacts to the other gender. 

Furthermore, it may be assumed that a high education level stands in a connection with a critical body perception and that social stronger families with higher incomes could realize their wish of change rather than lower class families. The last question of this survey (“The higher the costs for the desires operation, the lower improbable is it that a patient undergoes an operation”) seemed to be clear before the beginning of this survey. Interestingly the data rejected the hypothesis: In the here presented results, the amount of the costs stood not in a significant connection with the decision against a plastic-surgery intervention. 

An interpretation attempt for this result could be that people who are willing to undergo an operation accept the costs, if they suffer highly from an ugly part of their body. Perhaps high costs appear as a guarantee for qualified medical specialists, a professional execution of the operation and a successful result. For many people the wish for individual perfection of their body is priceless and therefore consequently has a greater value than the concern about financing of an intervention. A survey of the German Association for Aesthetic Plastic Surgery in the year 2012 showed that 84.2% of 1,200 persons, who underwent a surgery-intervention, based on aesthetic wishes. Only 7.2% indicated to undergo an operation by medical reasons. This suggest that the costs for or against an operation often play a secondary role. Last not least, from an attractive body new attitude towards life, new friendships or partnerships and even new jobs could develop from feeling to be beauty.
